# Stress-related cardiomyopathies

**DOI:** 10.1186/2110-5820-1-39

**Published:** 2011-09-20

**Authors:** Christian Richard

**Affiliations:** 1 AP-HP, Hôpital de Bicêtre, service de réanimation médicale, Le Kremlin-Bicêtre, F-94270 France; 2 Univ Paris-Sud, Faculté de médecine Paris-Sud, EA 4046, Le Kremlin-Bicêtre, F-94270 France

## Abstract

Stress-related cardiomyopathies can be observed in the four following situations: Takotsubo cardiomyopathy or apical ballooning syndrome; acute left ventricular dysfunction associated with subarachnoid hemorrhage; acute left ventricular dysfunction associated with pheochromocytoma and exogenous catecholamine administration; acute left ventricular dysfunction in the critically ill. Cardiac toxicity was mediated more by catecholamines released directly into the heart via neural connection than by those reaching the heart via the bloodstream. The mechanisms underlying the association between this generalized autonomic storm secondary to a life-threatening stress and myocardial toxicity are widely discussed. Takotsubo cardiomyopathy has been reported all over the world and has been acknowledged by the American Heart Association as a form of reversible cardiomyopathy. Four "Mayo Clinic" diagnostic criteria are required for the diagnosis of Takotsubo cardiomyopathy: 1) transient left ventricular wall motion abnormalities involving the apical and/or midventricular myocardial segments with wall motion abnormalities extending beyond a single epicardial coronary artery distribution; 2) absence of obstructive epicardial coronary artery disease that could be responsible for the observed wall motion abnormality; 3) ECG abnormalities, such as transient ST-segment elevation and/or diffuse T wave inversion associated with a slight troponin elevation; and 4) the lack of proven pheochromocytoma and myocarditis. ECG changes and LV dysfunction occur frequently following subarachnoid hemorrhage and ischemic stroke. This entity, referred as neurocardiogenic stunning, was called neurogenic stress-related cardiomyopathy. Stress-related cardiomyopathy has been reported in patients with pheochromocytoma and in patients receiving intravenous exogenous catecholamine administration. The role of a huge increase in endogenous and/or exogenous catecholamine level in critically ill patients (severe sepsis, post cardiac resuscitation, post tachycardia) to explain the onset of myocardial dysfunction was discussed. Further research is needed to understand this complex interaction between heart and brain and to identify risk factors and therapeutic and preventive strategies.

## Introduction

Neurocardiology has many dimensions, namely divided in three categories: the heart's effects on the brain (i.e., embolic stroke); the brain's effects on the heart (i.e., neurogenic heart disease); and neurocardiac syndromes, such as Friedreich disease [1]. The present review will focus on the nervous system's capacity to injure the heart. The relationship between the brain and the heart, i.e., the brain-heart connection, is central to maintain normal cardiovascular function. This relationship concerns the central and autonomic nervous systems, and their impairment can adversely affect cardiovascular system and induce stress-related cardiomyopathy (SRC) [2]. Even if it is unclear whether myocardial adrenergic stimulation is the only pathophysiological mechanism associated with SRC, enhanced sympathetic tone inducing endogenous catecholamine's stimulation of the myocardium was always reported [3].

The first description of suspected SRC was reported by W.B. Cannon in 1942 cited by Engel et al. [4] who published a paper entitled "Voodoo death," which reported anecdotal experiences of death from fright. This author postulated that death can be caused by an intense action of the sympathico-adrenal system. In 1971, Engel et al. collected more than 100 accounts from the lay press of sudden death attributed to stress associated with disruptive life events and provided a window into the world of neurovisceral disease (i.e., psychosomatic illness).

It is now widely admitted that this autonomic storm, which results from a life-threatening stressor, can be observed in the four following situations that induce left ventricle (LV) dysfunction [2]:

- Takotsubo cardiomyopathy or apical ballooning syndrome [5]

- Acute LV dysfunction associated with subarachnoid hemorrhage [6]

- Acute LV dysfunction associated with pheochromocytoma and exogenous catecholamine administration [7]

- Acute LV dysfunction in the critically ill [8]

## Brain-heart connection

Emotional and physical stress can induce an excitation of the limbic system. Amygdalus and hippocampus are, with the insula the principle brain areas, implicated in emotion and memory [9,10]. These areas play a central role in the control of cardiovascular function [9,10]. Their excitation provokes the stimulation of the medullary autonomic center, and then the excitation of pre- and post-synaptic neurons leading to the liberation of norepinephrine and its neuronal metabolites [11]. Adrenomedullary hormonal outflows increase simultaneously and induce the liberation of epinephrine. Epinephrine released from the adrenal medulla and norepinephrine from cardiac and extracardiac sympathetic nerves reach heart and blood vessel adrenoreceptors [1,9,10]. The occupation of the cardio-adrenoreceptors induces catecholamine toxicity in the cardiomyocytes [11].

Wittstein et al. compared plasma catecholamine levels in patients with SRC to those observed in patients with Killip class III myocardial infarction [3]. They reported a neurally induced exaggerated sympathetic stimulation in patients with SRC [3]. Thus a significant increase in plasma epinephrine, norepinephrine, dihydroxyphenylalanine, dihydroxyphenylglycol, and dihydroxyphenylacetic acid was observed and was consistent with the presence of enhanced catecholamine synthesis, neuronal reuptake, and neuronal metabolism, respectively [3] (Table 1). A significant increase in neuropeptide Y, which is stored in postganglionic sympathetic nerves, was observed in patients with SRC. By contrast the increase in plasma levels of metanephrine and normetanephrine, which are extra neuronal catecholamine metabolites, was within a similar range to that observed in Killip class III myocardial infarction patients [3]. This finding suggests that cardiac toxicity was mediated more by catecholamines released directly into the heart via neural connection than by those reaching the heart via the bloodstream.

**Table 1:** Plasma catecholamine levels in 13 patients with stress-related cardiomyopathy (Takotusbo) compared to 7 patients with Killip Class III myocardial infarction

Catecholamines (pg/ml)	Takotusbo (n = 13)	Infarctus Killip III (n = 7)	*p*	Normal value
Dihydroxyphénylalanine	2859 (2721- 2997)	1282 (1124-1656)	< 0.05	1755
Epinephrine	1264 (916-1374)	376 (275- 476)	< 0.05	37
Norepinephrine	2284 (1709-2910)	1100 (914- 1320)	< 0.05	169
Dopamine	111 (106- 146)	61 (46-77)	< 0.05	15

Median and interquartile range (25-75%). Mann-Whitney test. From reference (3) with permission.

The mechanisms underlying the association between this generalized autonomic storm secondary to a life-threatening stress and myocardial toxicity are widely discussed. Three mechanisms have been reported. Some authors have suggested that multivessel epicardial coronary artery spasm could supervene, but angiographic evidence of epicardial spasm was not reported by Wittstein et al. [3]. Coronary microvascular impairment resulting in myocardial stunning was suspected by some authors [12]. The most widely accepted mechanism of catecholamine mediated myocardial stunning is direct myocardial toxicity [13]. Catecholamines can decrease the viability of cardiomyocytes through cyclic AMP-mediated calcium overload and oxygen-derived free radicals [14]. This hypothesis was sustained by the myocardial histological changes observed in heart from patients suffering from SRC [1]. These histological changes are the same that those observed following high doses catecholamine infusion in animals. These changes differ from those observed in ischemic cardiac necrosis. Contraction band necrosis, neutrophil infiltration, and fibrosis reflecting high intracellular concentrations of calcium are generally observed [1]. It is now generally assumed that this calcium overload produces the ventricular dysfunction in catecholamine cardiotoxicity. The low incidence of the onset of these SRC and their description frequently reported in postmenopausal women suggested the possibility of a genetic predisposition [15,16]. Thus, Spinelli et al. evaluated the incidence of common polymorphisms of beta 1 and beta 2 adrenergic receptors, the Gs to which the receptors are coupled and GRK5 which desensitizes them [16]. They observed that the GRK5 Leu41 polymorphism was significantly more common in SRC than in a control group and suggested that this polymorphism was associated with an enhanced beta adrenergic desensitization which may predispose to cardiomyopathy caused by repetitive catecholamine surges [15,16].

## Stress related cardiomyopathies

### Takotsubo cardiomyopathy or apical ballooning syndrome

**Figure 1:**
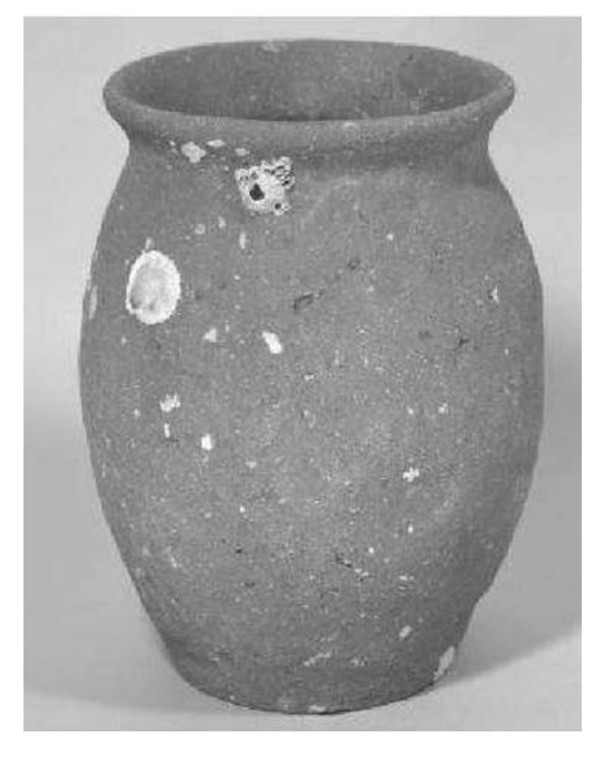
**The name Takotsubo was taken from the Japanese name for an octopus trap, which mimics the typical apical ballooning aspect of the left ventricle during the systole**.

Japanese authors reported in the nineties the first cases of reversible cardiomyopathy precipitated by acute and severe emotional stress in postmenopausal women [11,17-20]. This SRC was characterized by the onset of an acute coronary syndrome associated with a specific and reversible apical and wall motion abnormality despite the lack of coronary artery disease [11]. Initially, this syndrome was given the name Takotsubo cardiomyopathy and was secondarily referred to as the apical ballooning syndrome and broken heart disease [11,17-20]. The name Takotsubo was taken from the Japanese name for an octopus trap, which mimics the typical apical ballooning aspect of the left ventricle during the systole (Figure 1). Takotsubo has been reported all over the world and has been acknowledged by the American Heart Association and the American College of Cardiology as a form of reversible cardiomyopathy [21,22]. It has been estimated that 4-6% of women presenting with acute coronary syndrome suffered from Takotsubo [21].

**Figure 2:**
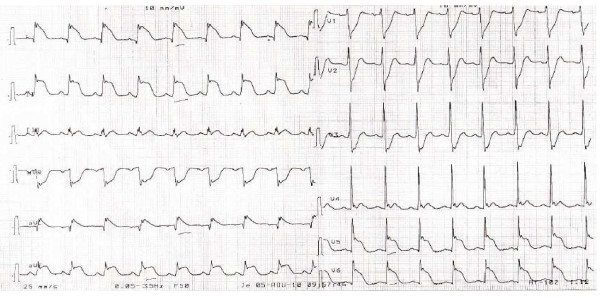
**Acute coronary syndrome with typical chest pain seen in a 62 years woman following emotional stress (death of a family member)**. Typical ST segment elevation. Echocardiography showed apical and mid ventricular wall motion abnormalities and hyperkinesis of the basal segment. Coronary angiography was normal. Cardiogenic shock supervened and needed circulatory assistance. Secondary favorable outcome. Introduction of beta-blockers after the correction of acute heart failure.

Usually seen in postmenopausal women, the clinical presentation of Takotsubo is similar to that of an acute coronary syndrome with typical chest pain and ECG abnormalities. Reported emotional stress included for example death of a family member, traffic road accidents, financial loss, and disasters, such as earthquakes [5,23,24]. In some patients, no clear precipitating factor can be identified. ST segment elevation on the ECG was observed in the majority of cases (Figure 2). Twenty-four to 40 hours later, T wave inversion supervened and q waves were seen in one third of the patients. Thus, there are no ECG criteria to discriminate between Takotsubo and acute myocardial infarction [5,23,24]. The elevation in troponin is very limited far from the huge increase observed during myocardial infarction. A very low incidence of in hospital mortality was reported, and heart failure, cardiogenic shock, and ventricular arrhythmias are observed in a minority of patients [11,17,23,25].

Typically, echocardiography showed apical and midventricular wall motion abnormalities and hyperkinesis of the basal myocardial segments [2]. These wall motion abnormalities did not correspond to a single epicardial coronary distribution. Apical and midventricular wall motion abnormalities can induce a dynamic obstruction in the LV outflow associated with a systolic anterior motion of the mitral leaflet.

**Figure 3:**
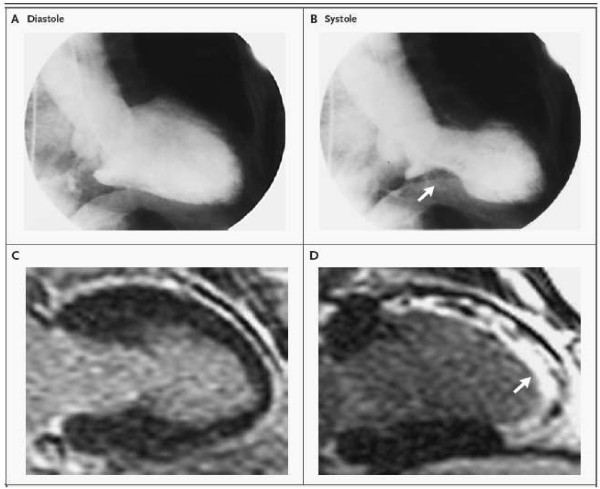
**Left ventricle angiography during diastole (A) and systole (B) showing apical and mid ventricular wall motion abnormalities and hyperkinesis of the basal segment (arrow)**. MRI in long axis showing that the akinetic regions are hypoenhanced and dark suggesting the presence of viable myocardium (**C**). Reference after an acute myocardial infarction showing hyperenhancement indicative of necrosis. From reference (3) with permission.

When performed, LV angiography confirmed these wall motion abnormalities (Figure 3) with the classical aspect of Takotsubo. Coronary angiography revealed the absence of obstructive epicardial coronary artery disease. Scintigraphic imaging and cardiac magnetic resonance imaging failed to reveal myocardial necrosis. Late gadolinium enhancement during cardiac magnetic resonance was absent eliminating ischemic myocardial necrosis [2]. Cardiac positron emission tomography using 18-fluorodeoxyglucose suggested an aspect of metabolic stunned myocardium associated with catecholamine excess. This stunned myocardium could be the consequence either of an intramyocardial calcium overload or ischemic-reperfusion phenomena [12-14].

Many morphological LV variants of Takotsubo have been reported: isolated midventricular and basal dysfunction with apical sparing, isolated basal hypokinesis, named inverse Takotsubo [11,26]. The reason for this noncoronary distribution of the segmental wall motion abnormalities was unknown and often related to differences in myocardial autonomic innervation and adrenergic stimulation [2,3,18].

Bybee and Prasad suggested four "Mayo Clinic" diagnostic criteria for Takotsubo: 1) transient LV wall motion abnormalities involving the apical and/or midventricular myocardial segments with wall motion abnormalities extending beyond a single epicardial coronary artery distribution; 2) absence of obstructive epicardial coronary artery disease that could be responsible for the observed wall motion abnormality; 3) ECG abnormalities, such as transient ST-segment elevation and/or diffuse T-wave inversion associated with a slight troponin elevation; and 4) the lack of proven pheochromocytoma and myocarditis [2].

Patients with suspected and/or proved Takotsubo must be monitored in intensive care. Because massive catecholamine release was observed in Takotsubo-induced stunned myocardium, beta agonists and vasopressors might be avoided whenever possible even in acute circulatory failure and mechanical circulatory support preferred if necessary. Sympathetic activation suggested the use of beta blocker therapy as soon as LV failure was corrected. The presence of a dynamic obstruction in the LV outflow precluded the initiation of an angiotensin-converting enzyme inhibitor, angiotensin receptor blocker, or diuretic treatment because of a possible potentiation. Anticoagulation with heparin was required to prevent left ventricle thrombus formation [18,24,27].

Echocardiographic examination will be regularly performed after hospital discharge to evaluate the resolution of LV dysfunction, which is complete in the majority of the patients after 1 to 3 months. A favorable prognosis has been widely reported in the more recent literature [23].

### Acute LV dysfunction associated with subarachnoid haemorrhage

ECG changes and LV dysfunction occur frequently after subarachnoid hemorrhage and ischemic stroke. This entity, referred as neurocardiogenic stunning, was called neurogenic SRC [2]. Four independent predictors of neurogenic SRC have been reported previously: severe neurologic injury, plasma troponin increase, brain natriuretic peptide elevation, and female gender [28]. The diagnosis of neurogenic SRC was associated with the potential onset of fatal arrhythmias and an increased risk of cerebral vasospasm. QT interval prolongation, ST segment elevation, and symmetrical T-wave inversion associated with an increase in cardiac troponin were observed in approximately two thirds of patients with severe subarachnoid hemorrhage [2]. As in the case of Takotusbo, neurogenic SRC often is difficult to distinguish from acute myocardial infarction. A slight increase in cardiac troponin and the onset of noncoronary distributed wall motion abnormalities suggest more a neurogenic SRC than an acute myocardial infarction.

Echocardiography shows hypokinesis involving basal and midventricular portion of the left ventricle, i.e., inverse Takotusbo. These findings are more usual than those observed in patients suffering from Takotusbo. Bybee and Prasad have suggested an algorithm for the evaluation of patients with subarachnoid haemorrhage and LV dysfunction associated with ECG abnormalities [2]. Similarities exist between Takotusbo and neurogenic SRC, which are both catecholamine-mediated. This suggests the existence of an overlap between these two entities [3]. Neurogenic SRC also was reported in patients with ischemic stroke and severe head trauma.

### Acute LV dysfunction associated with pheochromocytoma and exogenous catecholamine administration

LV dysfunction has been reported in the case of endogenous or exogenous over production of catecholamines. Pheochromocytoma is a rare neuroendocrine tumor located in the adrenal medulla that secretes catecholamines and particularly norepinephrine. Many case reports have suggested the onset of reversible LV dysfunction mimicking neurogenic SRC and rarely Takotusbo [7,26]. This LV dysfunction was reported during the catecholamine crisis and generally resolved after the surgical procedure [7,26]. Some case reports suggested that the administration of inhaled and/or intravenous exogenous catecholamines in patients with severe asthma and bronchospasm could be involved in the onset of transient neurogenic SRC [29]. Intracellular myocytes calcium overload due to catecholamine enhancement has been observed in myocardial biopsy specimens [30].

### Acute LV dysfunction in the critically ill

Acute LV failure occurs in approximately one-third to one-half of critically ill hospitalized patients. As reported by Chockalingam et al., determination as to whether the LV dysfunction is the cause, effect, or a coincidental finding has to be made and revisited periodically [8]. One of the most widely observed findings in critically ill patients is the onset of a global LV dysfunction. In patients with hemodynamic instability and acute circulatory failure, routine echocardiography is increasingly performed to exclude valvular heart disease, pericardial effusion, and acute coronary syndrome- related regional wall motion abnormalities.

If a previously undiagnosed dilated cardiomyopathy is excluded, global LV dysfunction can be partly explained by a relative contribution of direct catecholamine myocardial toxicity in the following situations: tachycardia-induced cardiomyopathy, hypertensive crisis, sepsis, multiorgan dysfunction, and postcardiac arrest syndrome. In these situations, a high incidence of myocardial injury assessed by cardiac troponin I levels was demonstrated despite the lack of acute coronary syndromes on admission to the intensive care unit [31,32]. Quenot et al. demonstrated that this myocardial injury was an independent determinant of in-hospital mortality even when adjusted for the SAPS II score [32].

#### Tachycardia-induced cardiomyopathy

Tachycardia-induced cardiomyopathy has been defined as a global systolic LV dysfunction secondary to atrial or ventricular tachyarrhythmias that reversed with rhythm control [33,34]. Studies in animals have suggested that the progression and the severity of heart failure were linked to the cadence of the heart rate, the duration of the tachycardia, and its cause. Thyroid dysfunction, dyskaliemia, hypoxia, and beta1-cardiac receptor stimulation may exacerbate this catecholamine storm. LV function normalized in a few days to weeks after the reduction of arrhythmias [33,34].

#### Hypertensive LV dysfunction

Mild troponin elevations, ischemic ECG changes, and LV dysfunction can be observed in patients with uncontrolled hypertension, for example, in patients suffering from neuroendocrine tumors, such as pheochromocytoma. Rapid blood pressure lowering was required with vasodilators, i.e., nitroglycerin infusions and/or oral administration of ACE inhibitors and angiotensin receptor antagonists, to prevent the onset of acute LV dysfunction and cardiogenic shock [8,35,36].

#### Sepsis and septic shock

Myocardial dysfunction, which is characterized by transient biventricular impairment of myocardial contractility, is commonly observed in patients suffering from severe sepsis and septic shock [37,38]. LV dysfunction has been associated with the elevation of cardiac troponin levels and indicated a poor prognosis in septic critically ill patients [8,31,32,37,39]. This elevation of the troponin levels occurred in the absence of flow limiting coronary artery disease. The transient increase in the troponin levels was probably the consequence of a loss of cardiomyocytes membrane integrity with a subsequent troponin leakage [8,31,32,37,39]. The mechanisms responsible for increase troponin levels and LV dysfunction are not clearly understood. The implication of systemic inflammatory response with the liberation of tumor necrosis factor alpha (TNF alpha) and other cardiosuppressive cytokines, such as interleukin-6, has been previously reported [8,31,32,37,39]. Histopathological studies in patients with LV dysfunction and septic shock revealed contraction band necrosis previously reported in case of sympathetically mediated myocardial injury [40]. Moreover during severe sepsis, oxidative stress and oxygen free radicals could inactivate catecholamine by an enhancement of their transformation in adrenochromes [41]. The production of adrenochromes explains the loss of the vasoconstrictive effect of endogen and exogen catecholamines [41]. It also could partly explain myocardial toxicity and troponin liberation due to the loss of integrity of the membrane of cardiomyocytes [40]. This deactivation of the catecholamines suppresses their role in the inhibition of TNF alpha production, which is a well-known cardiosuppressive cytokine.

By contrast, some authors consider sepsis-induced myocardial depression an adaptative and at least partially protective process [42,43]. They have suggested that the myocardial depression was the consequence of the attenuation of the adrenergic response at the cardiomyocyte level due to down-regulation of the beta adrenergic receptors and depression of the postreceptor signaling pathways [42,43]. This hibernation-like state of the cardiomyocytes during severe sepsis was probably enhanced by neuronal apoptosis in the cardiovascular autonomic centers and by inactivation of catecholamines secondary to the production of reactive oxygen species by oxidative stress [44]. This physiopathological approach is reinforced by the potential harmful effect of all strategies designed to enhance oxygen delivery above supranormal values by inotropes and vasoconstrictors [45].

Thus, to keep adrenergic stimulation of the heart at the minimum level, some recently published papers suggested a place for beta-blockers to favor the enhancement of the decatecholaminization in septic critically ill patients [42,43,46]. Obviously, the titration of an adequate dosage of beta-blockers for these hemodynamically unstable patients is difficult to find during the acute phase. However, as in patients with SRC, the administration of beta-blockers as soon as possible after stabilization of the circulatory failure might be suggested or at least investigated in prospective, randomized, clinical studies [42,43,46]. Recent data suggest that beta-blockers exert favorable effects on metabolism, glucose homeostasis, and cytokine expression in patients with severe sepsis [47]. It has been reported that septic patients hospitalized in critical settings, previously treated with beta-blockers, have a better outcome [37,42,43,46,47].

#### Postcardiac arrest myocardial dysfunction

Prengel et al. reported that severe stress, such as that occurring with cardiac arrest and cardiopulmonary resuscitation, activates the sympathetic nervous system and causes a rise in plasma catecholamine concentrations, which could play a role in the onset of post cardiac arrest myocardial dysfunction [48]. This postcardiac arrest myocardial dysfunction contributes with postcardiac arrest brain injury to the low survival rate after in- and out-of-hospital cardiac arrest [48,49]. However, this myocardial dysfunction is responsive to therapy and reversible, suggesting a stunning phenomenon rather than a permanent and irreversible myocardial injury (i.e., myocardial infarction) [50].

The time to recovery appeared to be between 24 and 48 hours and complete for a wide majority of the patients. Laurent et al. reported that cardiac arrest survivors have reduced cardiac output 4 to 8 hours later [50]. Cardiac output improved substantially by 24 hours and almost returned to normal by 72 hours in patients who survived out-of-hospital cardiac arrest. Using multivariate analysis, Laurent et al. demonstrated that the amount of epinephrine used during cardiopulmonary resuscitation predicted the occurrence of hemodynamic instability [50]. These results confirm experimental data that suggest that epinephrine potentiates myocardial dysfunction after resuscitation [51]. Previous clinical studies suggest that high doses of epinephrine infused during resuscitation may alter the cardiac index after return of spontaneous circulation and could be an independent predictor of mortality [52]. Many experimental studies reported that epinephrine, when administered during cardiopulmonary resuscitation, significantly increased the severity of post resuscitation myocardial dysfunction as a consequence of its beta_1_-adrenergic actions [50-52]. This result was associated with significantly greater postresuscitation mortality. Thus, it would be appropriate to reevaluate epinephrine as the drug of first choice for cardiac resuscitation.

In conclusion, SRC can occur after an acute physical or psychological stress, subarachnoid hemorrhage, pheochromocytoma crisis, acute medical illness, such as severe sepsis, and after the administration of exogenous catecholamine administration. The presence of contraction band necrosis in the myocardial biopsy specimen suggests a catecholamine-mediated mechanism even if other pathophysiological mechanisms have been suggested. Further research is needed to understand this complex interaction between heart and brain and to identify risk factors and therapeutic and preventive strategies.

## Competing interests

The author declares that they have no competing interests.
